# p62 functions as an oncogene in colorectal cancer through inhibiting apoptosis and promoting cell proliferation by interacting with the vitamin D receptor

**DOI:** 10.1111/cpr.12585

**Published:** 2019-02-22

**Authors:** Jing Zhang, Suzhen Yang, Bing Xu, Ting Wang, Ying Zheng, Fei Liu, Fenggang Ren, Jiong Jiang, Haitao Shi, Baicang Zou, Xiaolan Lu, Shemin Lu, Lei Dong

**Affiliations:** ^1^ Department of Digestive Disease and Gastrointestinal Motility Research Room The Second Affiliated Hospital of Xi’an Jiaotong University Xi’an China; ^2^ Department of Digestive Disease and Gastrointestinal Motility Research Room Xi’an Jiaotong University Xi’an China; ^3^ Clinical Research Center of Shanxi Province for Dental and Maxillofacial Diseases, College of Stomatology Xi'an Jiaotong University Xi’an China; ^4^ National Local Joint Engineering Research Center for Precision Surgery and Regenerative Medicine Xi’an Jiaotong University Xi’an China; ^5^ Department of Digestive Disease Shanghai Pudong Hospital, Fudan Affiliated Pudong Medical Center Shanghai China; ^6^ Department of Biochemistry and Molecular Biology, School of Basic Medical Sciences Xi'an Jiaotong University Health Science Center Xi’an China

**Keywords:** apoptosis, colorectal cancer, proliferation, SQSTM1/p62, vitamin D receptor

## Abstract

**Objectives:**

The role of p62 in cancer is controversial. Evidence has shown that p62 is upregulated in different cancers and promotes tumour growth, such as in liver cancer and lung cancer. However, a recent study showed that the downregulation of p62 in hepatic stellate cells (HSCs) promotes hepatocellular carcinoma (HCC) development. How p62 is regulated in colorectal cancer (CRC) remains largely unknown. In this study, we aimed to investigate the roles and molecular mechanisms of p62 in CRC.

**Materials and Methods:**

The expression levels of p62 in CRC tissues and adjacent non‐tumour tissues were determined by immunohistochemistry (IHC). Stable p62‐overexpression HCT116 cells and p62‐knockdown SW480 cells were established with lentiviral vectors. The role of p62 in CRC was investigated in in vitro and in vivo functional studies. The relationship between p62 and the vitamin D receptor (VDR) was investigated by coimmunoprecipitation (Co‐IP) assays.

**Results:**

p62 was significantly upregulated in CRC, and a high p62 level was an independent risk factor for a poor prognosis in CRC patients. p62 promoted CRC migration and invasion by inhibiting apoptosis and promoting cell proliferation in vitro, and p62 aggravated tumour growth and metastasis in vivo. Co‐IP assays indicated that p62 interacts with the VDR and may target the NRF2‐NQO1 axis.

**Conclusions:**

Our study suggested that p62 functions as an oncogene in CRC through inhibiting apoptosis and promoting cell proliferation by interacting with the VDR.

## 1 INTRODUCTION

Colorectal cancer (CRC) is one of the deadliest cancers worldwide and develops by the accumulation of genetic and environmental factors.^1^ In 2012, the International Agency for Research on Cancer (IARC) has reported that CRC is the third most common cancer in men (10.0% of global incident cancer cases in men) and the second most common cancer in women (9.2% of global incident cancer cases in women). The mortality of CRC is the third highest in all cancers, bringing a great burden to human beings,^2^ but the pathogenesis of CRC remains unclear.

Protein p62 is a classic autophagy receptor and encodes the gene *SQSTM1*. It has four structural domains, including LIR (LC3‐interacting region), PB1 (Phox/Bem 1p), TB (TRAF6‐binding) and UBA (ubiquitin‐associated) domains. The LIR domain is combined with the autophagy regulator ATG8/LC3.^3^ Autophagy can be activated by metabolism in an emergency. When cancer cells are unable to obtain extracellular metabolites and energy sources, autophagy may promote tumour development.^4^, ^5^ As a ubiquitin‐binding protein, p62 plays an important role in response to DNA damage. Autophagy‐deficiency‐induced p62 accumulation can inhibit DNA double‐strand break (DSB)‐induced histone and chromatin ubiquitination.^6^ The role of p62 in cancer is controversial. Evidence has shown that p62 is upregulated in different cancers and promotes tumour growth, such as in liver cancer^7^ and lung cancer.^8^ The accumulation of p62 enhanced endoplasmic reticulum (ER) stress, mitochondrial dysfunction, reactive oxygen species (ROS) production and genome damage. The inhibition of p62 can suppress tumorigenesis.^6^ However, a recent report from Duran et al adds a series of interesting twists to the role of p62 in cancer. They found that p62 inhibits the activity of hepatic stellate cells (HSCs) by promoting the formation of the vitamin D receptor (VDR)‐retinoid X receptor (RXR) heterodimerization and that the downregulation of p62 in HSCs promotes HCC development by reducing the interaction of VDR‐RXR.^9^

Vitamin D is widely known for its role in calcium and phosphate metabolism. In the mid‐1970s, scientists found that a nuclear receptor, VDR, was present in numerous tissues. It is not related to calcium and bone but associated with a large number of other diseases.^10^ Enhanced VDR signalling can reduce β cell failure and inhibit type 2 diabetes.^11^ VDR can reduce myocardial ischaemia/reperfusion injury by reducing oxidative stress and inhibiting apoptosis‐mediated cell death.^12^ Morphine‐induced T‐cell apoptosis is mediated through the downregulation of VDR and the activation of renin‐angiotensin system (RAS).^13^ In genistein, VDR can mediate the proliferation of MC3T3‐E1 cells but is not affected by the oestrogen receptor.^14^ Vitamin D is also important against bacterial infections. Bacteria can inhibit the expression of VDR and leucine‐leucine‐37 (LL‐37) to avoid antibacterial defence. Vitamin D supplementation can upregulate the expression of VDR and LL‐37 of macrophages and enhance the immunological defence against spontaneous peritonitis in patients with cirrhosis.^15^ A recent study examined the status of LL‐37, tumour necrosis factor (TNF) and vitamin D in tuberculosis (TB) patients. They find that the mean levels of LL‐37 and TNF were higher in the TB group compared with healthy controls. However, there was no correlation between levels of LL‐37, TNF and vitamin D in patients with TB.^16^ In tumours, high VDR expression in CRC stromal fibroblasts is associated with better overall survival.^17^ In addition, Vitamin D deficiency resists proliferation and promotes apoptosis though the downregulation of Wnt/β‐catenin signalling in human breast and prostate cancers.^18^

In this study, we aimed to investigate the roles and molecular mechanisms of p62 in CRC. We found that p62 is significantly upregulated in CRC, and p62 functions as an oncogene in CRC through inhibiting apoptosis and promoting cell proliferation by interacting with the VDR.

## 2 MATERIALS AND METHODS

### 2.1 Clinical samples and tissue microarray

Twenty‐two primary paired CRC tissues (adjacent non‐tumour tissues and CRC tissues) selected from the Second Affiliated Hospital of Xi'an Jiaotong University (Xi'an, China) were used for Western blot detection and real‐time quantitative PCR (RT‐qPCR). Before specimen collection, every patient gave informed consent, and the Clinical Research Ethics Committee of the Second Affiliated Hospital of Xi'an Jiaotong University approved the study.

Tissue microarrays (HColA180Su15) containing 80 adjacent non‐tumour tissues and 100 CRC tissues were purchased from Shanghai Outdo Biotech (Shanghai, China). The microarray tissues were from clinical phase 1, phase 2, phase 3 and phase 4. The operative time was from April 2008 to November 2008, and the follow‐up time was in July 2015. The follow‐up time interval was 6.7‐7.2 years.

### 2.2 Cell lines and cell culture

Human CRC cell lines HT29, HCT116, SW480, SW620, Caco2 and DLD‐1 and the normal human colon cell line HCoEpiC were cultured in high glucose DMEM containing 10% foetal bovine serum (FBS; Gemini, Calabasas, CA, USA). All cells were maintained in a humidified incubator in 5% CO_2_ at 37°C.

### 2.3 Lentiviral transduction

The hU6‐MCS‐CMV‐EGFP knockdown lentiviral vector for *SQSTM1*, the Ubi‐MCS‐3FLAG‐SV40‐puromycin overexpressing lentiviral vector for *SQSTM1* and the control lentiviral vector were obtained from Genechem Co., Ltd. (Shanghai, China). The SW480 p62‐knockdown cells using lentivirus infection and the efficiency of transduction were controlled by GFP fluorescence. A stable HCT116 p62‐overexpression cells was established using lentivirus infection and selected with 2 ng/mL puromycin.

### 2.4 Cell viability analysis

Cell viability was detected using the CCK‐8 assay. Cells were seeded in 96‐well plates at a density of 5 × 10^3^ cells in 100 μL of medium and cultured for 1‐4 days. Then, CCK‐8 reagent was added to each well. After an hour reaction at 37°C, the absorbance of the density of each well was read at a wavelength of 450 nm with a microplate reader (Thermo, Waltham, MA, USA).

### 2.5 Migration and invasion assays

Cell migration and invasion assays were evaluated using a Matrigel Invasion Chamber (Corning, Corning, NY, USA). For migration assays, 1.5 × 10^5^ cells were seeded into the upper chamber in 200 μL of serum‐free DMEM. Then, 700 μL of DMEM with 30% FBS was added to the lower chamber, and the cells were incubated for 36‐48 hours. For the invasion assay, the upper chambers were covered with 60 μL of Matrigel (200 mg/mL; BD Biosciences, Franklin lake, NJ, USA) and dried for 6 hours in an incubator. A total of 2.0 × 10^5^ cells were seeded into the upper chamber in 200 μL of serum‐free DMEM. Then, 700 μL of DMEM with 30% FBS was added to the lower chamber, and the cells were incubated for 48‐72 hours. Afterwards, cells in the upper chamber were removed, and cells that migrated/invaded through the pores were fixed in 100% methanol and stained with 0.5% crystal violet. The number of migrating/invading cells was counted with a microscope at 200× magnification in five random fields.

### 2.6 Wound healing

Cells were seeded into 6‐well plates. After confluence, cells were scratched using a 1 mm wide tip and cultured in serum‐free DMEM. Images were captured with a microscope at 100× magnification at 0, 24 and 48 hours. Wound spacing was calculated and analysed.

### 2.7 Colony formation

One thousand cells were seeded into 6‐well plates and incubated at 37°C for 14 days. Then, cells were fixed in 100% methanol and stained with 0.5% crystal violet, and colonies were counted.

### 2.8 Flow cytometry

Cell apoptosis was detected using a PE Annexin V/7‐amino‐actinomycin (PE/7‐AAD) Detection Kit (BD Biosciences) and analysed by flow cytometry.

### 2.9 Mouse xenograft and metastasis models

Male athymic nude mice (BALB/c, 5 weeks old) were purchased from the Xi'an Jiaotong University Medical Laboratory Animal Center. All experiments were approved by Xi'an Jiaotong University. For xenograft models, five nude mice in each group were subcutaneously injected with 1 × 10^6^ cells. After a month, the mice were sacrificed, and the tumours were weighed. For metastasis models, each group of mice was injected with 1 × 10^6^ cells in the tail vein and sacrificed after 2 months. Lung tissue was removed for HE staining.

### 2.10 Western blotting analysis

Total protein was isolated using RIPA buffer (Beyotime, Shanghai, China) with a protease‐inhibitor cocktail (Bimake, Houston, TX, USA). The proteins were separated by SDS‐PAGE and transferred onto PVDF membranes. The membranes were blocked with 10% milk for 2‐4 hours and incubated with primary antibodies at 4°C overnight. The primary antibodies used in the experiment were as follows: anti‐vimentin (1:1000; ab92547; Abcam, Cambridge, UK); anti‐E‐cadherin (1:1000; 3195; CST, Darmstadt, Germany); anti‐cleaved‐caspase‐7 (1:1000; 9491; CST); anti‐cleaved PARP1 (1:1000; ab32064; Abcam); anti‐*SQSTM1*/p62 (1:10000; ab109012; Abcam); anti‐VDR (1:1000; ab109234; Abcam); anti‐c‐Myc (1:1000; ab32072; Abcam); anti‐CDK2 (1:5000; ab32147; Abcam); anti‐cyclin D1 (1:10000; ab40754; Abcam); anti‐NQO1 (1:10000; ab80588; Abcam); anti‐Nrf2 (1:1000; ab62352; Abcam); anti‐GAPDH (1:1000; NC021; Zhuangzhi, Xi'an, China); anti‐β‐actin (1:1000; NC011; Zhuangzhi); and anti‐rabbit IgG, light chain specific (1:5000; SA00001‐7L; Proteintech, Chicago, IL, USA). After washing in TBST buffer (with 0.1% Tween 20), the membranes were incubated with the secondary anti‐rabbit IgG (1:5000; EK020; Zhuangzhi) for 2 hours. At last, the membranes were visualized by the GeneGnome XRQ System. In addition, anti‐SQSTM1/P62 (1:40; ab207305; Abcam), anti‐VDR (1:40; ab3508; Abcam), protein A and protein G magnetic beads (LSKMAGG02; Millipore, Boston, MA, USA) were used for Co‐IP.

### 2.11 RNA extraction and reverse transcriptase RT‐qPCR

Total RNA was extracted by TRIzol (Invitrogen, Thermo, Waltham, MA, USA). The Transcriptor First‐Strand cDNA Synthesis Kit (Roche, Basel, Denmark) was used for reverse transcription. RT‐qPCR analysis was performed with the FastStart Universal SYBR Green Master (Roche). Steps were performed according to the protocol described by the manufacturer. The 2^−ΔΔ^ Ct method was used to determine the relative quantitative gene expression levels with β‐actin as a reference. The primer sequences were as follows: β‐actin (forward 5′‐GGCACCACACCTTCTACAATGAGC‐3′, reverse 5′‐GATAGCACAGCCTGGATAGCAACG‐3′), SQSTM1 (forward 5′CCGTCTACAGGTGAACTCCAGTCC‐3′, reverse 5′‐AGCCAGCCGCCTTCATCAGAG‐3′).

### 2.12 Immunohistochemistry (IHC)

Paraffin‐embedded slides and tissue microarrays were dewaxed with dimethylbenzene (I and II) and gradient alcohol (100%, 95%, 85% and 75%). The antigen retrieval was performed by microwaving sections in citrate buffer for 2 minutes. Slides were incubated with 3% hydrogen peroxide (H_2_O_2_) for 20 minutes and then incubated with goat serum for 30 minutes at room temperature. Without washing, primary antibodies *SQSTM1*/p62 (1:1000; ab109012; Abcam), Ki67 (1:50; ab8191; Abcam) and caspase‐7 p20 (1:50; SC‐28295; Santa Cruz Biotechnology, Santa Cruz, DE, USA) were applied at 4°C overnight. Biotinylated anti‐IgG was added and incubated at room temperature for 1 hour. After incubating with streptomycin‐HRP for 30 minutes, the slides were stained with DAB and then counterstained with haematoxylin. After washing with flowing water, the slides were dehydrated with gradient alcohol and dimethylbenzene. Finally, the slides were sealed with neutral balsam and coverslips.

### 2.13 Evaluation of immunostaining intensity

Tissue microarrays were scored independently by two pathologists who were blinded to the clinicopathologic features and outcomes of the patients. The staining was evaluated under low magnification (40×), then selected five views under high magnification (200×) for analysis. Immunoreactivity was divided into five grades (percentage core) according to the percentage of stained cells: no staining (0), 1%‐25% (1), 26%‐50% (2), 51%‐75% (3) and >75% (4). The intensity of staining was divided into four grades (intensity scores): negative (0), weak (1), moderate (2) and strong (3). Then, a final histological overall score was calculated by the multiplication of the two values. An overall score of 0‐12 was calculated and graded as low (score < 7) or high (score ≥ 7).

### 2.14 Statistical analysis

graphpad prism 5 (La Jolla, CA, USA) was used for all statistical analyses. The comparisons between two groups were performed using Student's *t* test. Before using Student's *t* test, we performed a variance homogeneity test and normality test for the variable data. The overall survival rates were estimated using Kaplan‐Meier analyses and log‐rank tests. The chi‐squared test was used to determine whether there was a significant difference in the distribution of p62 samples among the different categories. A Cox regression model was used for univariate and multivariate analyses to calculate the hazard ratio and its 95% confidence interval. Data are presented as the mean ± SD. All statistical tests were two‐sided, and values of *P* < 0.05 were considered significantly different.

## 3 RESULTS

### 3.1 p62 is upregulated in CRC tissues and indicates poor prognosis in human CRC

**Figure 1:**
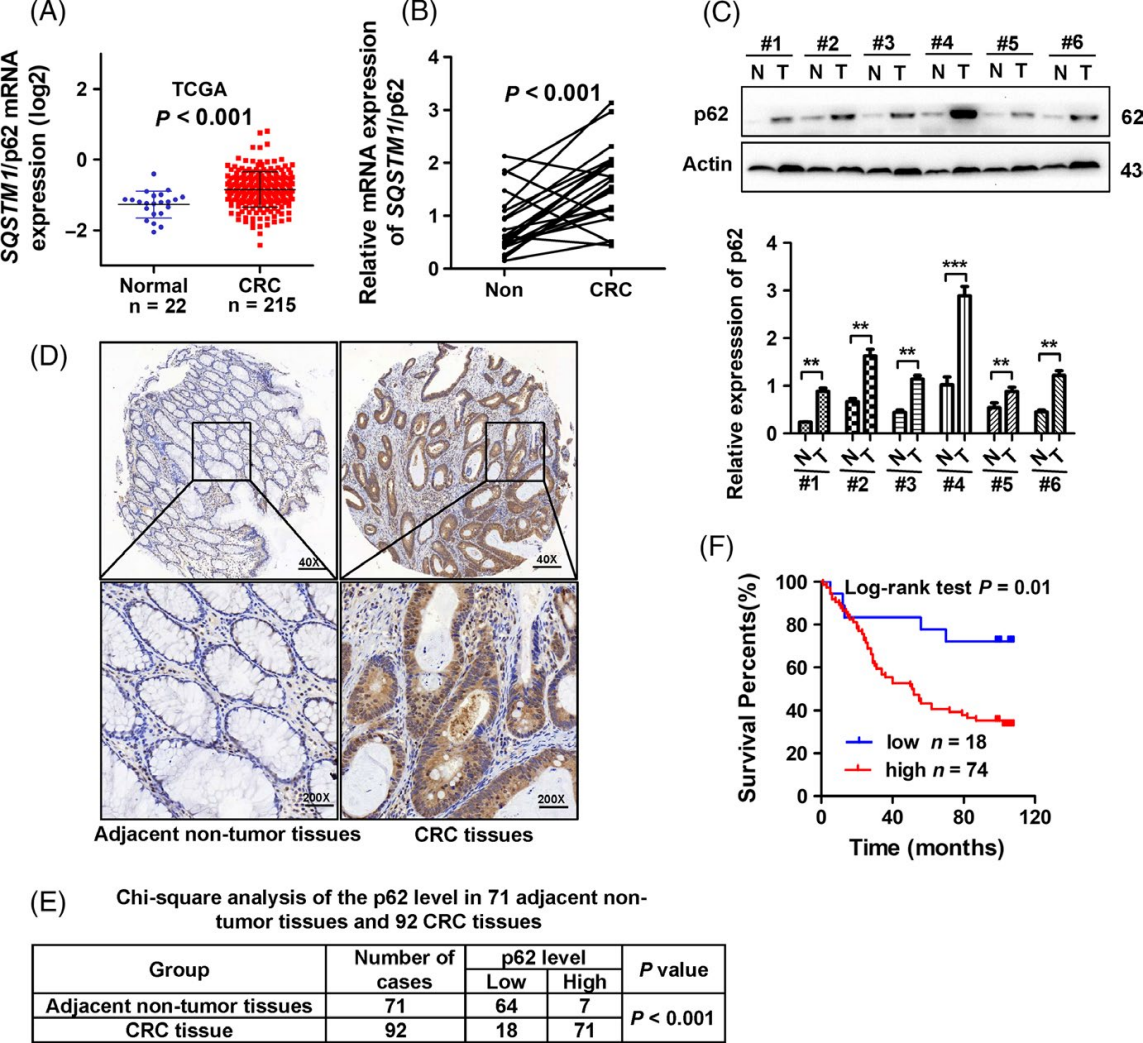
p62 is upregulated in CRC tissues and indicates poor prognosis in human CRC. A, *SQSTM1/*p62 mRNA expression levels are upregulated in CRC tissues compared to normal controls in the TCGA dataset (Student's *t* test, *P* < 0.001). B, Relative mRNA expression of p62 in primary CRC tissues and paired adjacent non‐tumour tissues (Non) (n = 22), (Student's *t* test, *P* < 0.001). C, Western blot analysis of p62 in 6 representative paired adjacent non‐tumour tissues (N) and CRC tissues (T). Bars represent the standard error of the mean ± SD from three independent experiments (Student's *t* test, **P < 0.01). D, Representative images of IHC staining of p62 in 92 CRC tissues and 71 adjacent non‐tumour tissues. The high (right) and low (left) expression levels of p62 were evaluated semi‐quantitatively by the staining intensity (high score: 7‐12; low score: 0‐6). E, Chi‐square analysis of the p62 levels in 92 CRC tissues and 71 adjacent non‐tumour tissues (*P* < 0.001). F, Kaplan‐Meier curve depicting the overall survival of the CRC patients (n = 92). The curves were stratified based on the p62 level (log‐rank test, *P* = 0.01)

We first investigated the involvement of p62 in CRC patients from the TCGA dataset and identified that *SQSTM1*/p62 mRNA expression was significantly increased in CRC tissues compared to normal controls (Figure 1A, *P* < 0.001). We also examined the expression of p62 in 22 pairs of human CRC tissues and adjacent non‐tumour tissues. The mRNA expression of *SQSTM1*/p62 was upregulated in 18 out of 22 (81.8%) human CRC tissues compared with the adjacent non‐tumour tissues as determined by RT‐qPCR (Figure 1B, *P* < 0.001). Consistently, p62 protein expression was upregulated in CRC tissues compared to the paired adjacent non‐tumour tissues as indicated by Western blotting (Figure 1C, *P* < 0.01). Moreover, IHC staining analysis showed higher expression of p62 in CRC tissues compared with the adjacent non‐tumour tissues (Figure 1D,F, *P* < 0.001). Higher p62 expression was not correlated with gender, age, tumour location, or size (Table 1, *P* > 0.05) but was significantly positively associated with lymph node metastasis and American Joint Committee on Cancer (AJCC) status (Table 1, *P* < 0.05). In addition, the patients with higher expression of p62 had shorter overall survival than those with lower expression of p62, indicating that the p62 level was an independent risk factor for a poor prognosis in CRC patients (Figure 1F, *P* = 0.01 and Table 2, *P* < 0.05).

**Table 1:** Prognostic factors in colon cancer patients by univariate analysis

Parameter	n	Cumulative survival rates (%)		Mean survival time (mo)	Hazard ratio	95% confidence interval	*P* value
		3‐y	5‐y				
Gender							
Male	52	63.5	42.3	65.442	1.121	0.655‐1.918	0.677
Female	40	57.5	50.0	62.356			
Age							
<60	20	70.0	60.0	74.1	1.585	0.775‐3.245	0.207
≥60	72	58.3	47.2	61.218			
Location							
Colon ascendens	33	48.5	45.5	55.242			0.276
Colon transversum	19	63.2	42.1	58.916	1.797	0.918‐3.515	0.087
Colon descendens	11	63.6	54.5	69.182	0.802	0.802‐3.635	0.165
Colon sigmoideum/rectum	29	72.4	58.6	77.00	0.371	0.371‐2.861	0.954
Tumour size							
<5 cm	35	71.4	48.6	66.8	1.052	0.608‐1.819	0.857
≥5 cm	57	54.4	50.9	62.404			
Lymph node metastasis							
Negative	55	76.4	61.8	75.818	2.481	1.447‐4.256	0.001
Positive	37	37.8	32.4	46.014			
AJCC stage							
I	4	100	100	100.25			<0.001
II	50	84.0	60.0	71.98	0.00	0.00	0.970
III	36	38.9	33.3	46.67	0.029	0.005‐0.149	<0.001
IV	2	0	0	5.5	0.065	0.013‐0.329	0.001
P62 level							
Low	18	83.3	77.8	86.667	3.136	1.247‐7.889	0.015
High	74	55.4	43.2	58.660			
Overall	92	60.9	50.0	64.124			

**Table 2:** Multivariate analysis using the Cox proportional hazards model

Parameter	n	Hazard ratio	95% confidence interval	*P *value
Gender				
Male	52	1.002	0.544‐1.844	0.995
Female	40			
Age				
<60	20	1.696	0.797‐3.609	0.170
≥60	72			
Tumour size				
<5 cm	35	1.169	0.654‐2.088	0.599
≥5 cm	57			
Lymph node metastasis				
Negative	55	0.182	0.043‐0.774	0.021
Positive	37			
AJCC stage				
I	4	11.857	3.411‐41.217	<0.001
II	50			
III	36			
IV	2			
P62 level				
Low	18	2.723	1.070‐6.931	0.036
High	74			

### 3.2 p62 shows tumour‐promoting ability in CRC cells

**Figure 2:**
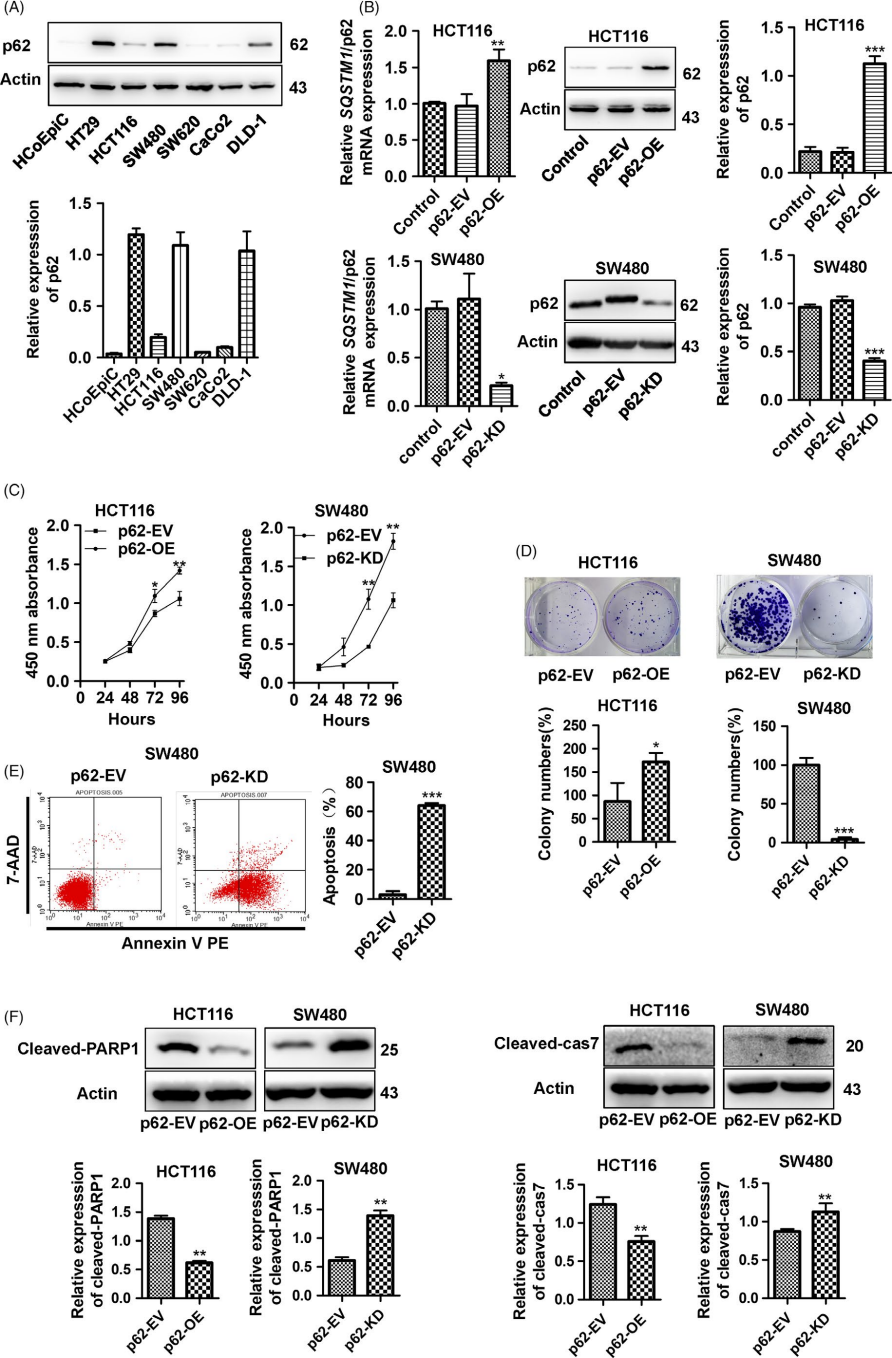
p62 shows tumour‐promoting ability in CRC cells. A, Western blotting of p62 in 6 human CRC cell lines and one normal human intestinal epithelial cell line. B, HCT116 was stably transduced with a p62‐overexpression lentiviral vector (p62‐OE), and SW480 was stably transduced with a p62‐knockdown lentiviral vector (p62‐KD). Accordingly, control groups were transduced with the corresponding lentiviral carrying an empty vector (EV). Overexpression or silencing of p62 was confirmed by RT‐qPCR (left panel) and Western blotting (right panel). C, Overexpression of p62 significantly promoted cell viability by CCK‐8 assay (left panel) compared to that of the control groups in HCT116. Knockdown of p62 significantly reduced cell viability by CCK‐8 assay (right panel) compared to that of the controls in SW480. D, Overexpression of p62 significantly increased colony numbers (left panel) compared to those of the control groups in HCT116. Knockdown of p62 significantly decreased colony numbers (right panel) compared to those of the controls in SW480. E, Apoptosis was analysed by flow cytometry. Knockdown of p62 in SW480 cells induced early apoptosis compared to the control cells. F, Expression of the cleaved form and the total level of caspase‐7 and PARP1 were detected by Western blotting. Bars represent the standard error of the mean ± SD from three independent experiments. *Represents Student's *t* test *P < 0.05, **P < 0.01 and ***P < 0.001.

The upregulation of p62 in clinical CRC tissues suggested the potential tumour‐promoting ability of p62 in the progression of CRC. To further evaluate the oncogenic role of p62 in CRC, we also explored the expression of p62 in 6 CRC cell lines, HT‐29, HCT116, SW480, SW620, Caco2, and DLD‐1, and the normal colonic epithelial cell line HCoEpiC. p62 was upregulated in HT29, SW480 and DLD‐1, while it was silenced in HCT116, SW620, Caco2 and the immortalized normal colonic epithelial cell line HCoEpiC (Figure 2A). Then, we established stable p62‐overexpression HCT116 cells and stable p62‐knockdown SW480 cells. The expression of p62 in HCT116‐p62 and SW480‐p62 cells was confirmed by RT‐qPCR and Western blotting (Figure 2B, *P* < 0.05). The overexpression of p62 in HCT116 cells significantly promoted cell growth compared with HCT116‐EV cells as determined by the CCK‐8 assay (Figure 2C, *P* < 0.05), while the knockdown of p62 in SW480 cells suppressed cell growth (Figure 2C, *P* < 0.01). The proliferation‐promoting effect of p62 was further confirmed by the increased colony formation efficiency in p62‐overexpression HCT116 cells compared with that of the HCT116‐EV cells (Figure 2D, *P* < 0.05) and the reduced colony formation ability in p62‐knockdown SW480 cells compared with that of the SW480‐EV cells (Figure 2D, *P* < 0.001). These data collectively suggest that p62 plays a tumour‐promoting role in CRC cells. In addition, the effect of p62 on cell apoptosis was evaluated. The knockdown of p62 in SW480 cells induced early apoptosis compared to the control cells (Figure 2E, *P* < 0.001). The overexpression of p62 in HCT116 cells suppressed early apoptosis compared to the control cells, but no significant difference was found (Figure S1, *P* > 0.05). The effect of p62 on cell apoptosis was further examined by the reduced protein expression of cleaved‐caspase‐7(cleaved‐cas7) and cleaved‐PARP1 in p62‐overexpression HCT116 cells and by the increased expression of these proteins in p62‐knockdown SW480 cells (Figure 2F, *P* < 0.01). These results indicated that the oncogenic effect of p62 is also associated with the suppression of cell apoptosis.

### 3.3 p62 promotes the invasion and migration abilities in CRC cell lines

**Figure 3:**
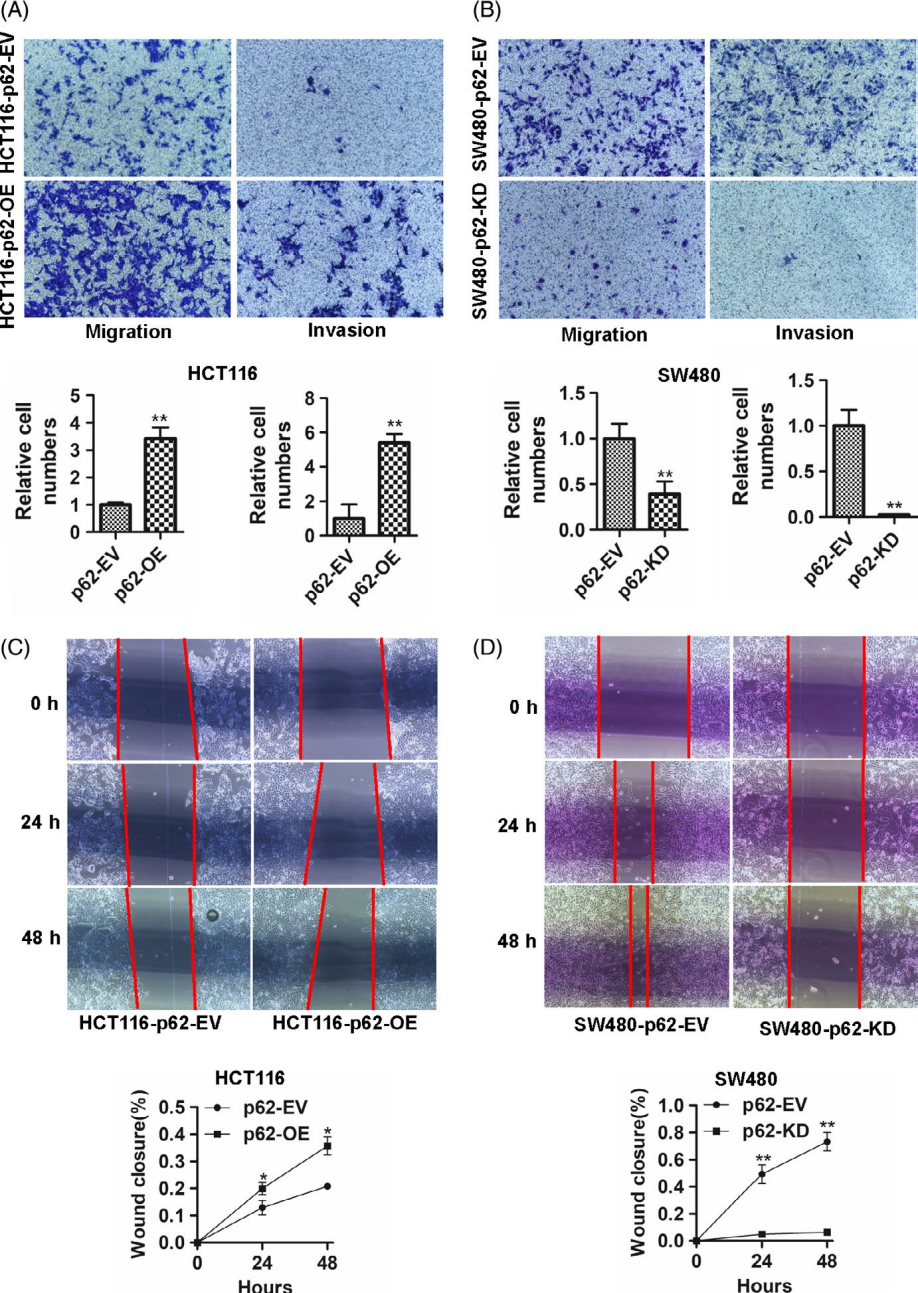
p62 promotes the invasion and migration abilities in CRC cell lines. A and B, Overexpression of p62 promoted invasion and migration abilities in HCT116 cells by transwell studies. Knockdown of p62 showed the opposite effect on cell invasion and migration in SW480 cells. C and D, Wound healing assays were used to evaluate the migration of the indicated cells. Bars represent the standard error of the mean ± SD from three independent experiments. *represents Student's *t* test *P < 0.05 and **P < 0.01

The oncogenic role of p62 in CRC was further evaluated for invasion and migration. Transwell assays showed that invasion and migration were significantly enhanced in p62‐overexpression HCT116 cells (Figure 3A, *P* < 0.01). Conversely, p62‐knockdown SW480 cells showed the opposite effect on cell invasion and migration (Figure 3B, *P* < 0.01). In addition, the monolayer scratch healing assays showed that the overexpression of p62 significantly increased the migration ability of HCT116 cells (Figure 3C, *P* < 0.05), while knockdown of p62 in SW480 cells suppressed the migration capacity (Figure 3D, *P* < 0.01), indicating that p62 enhances CRC invasion and migration abilities.

### 3.4 p62 aggravates tumour growth and metastasis in vivo

**Figure 4:**
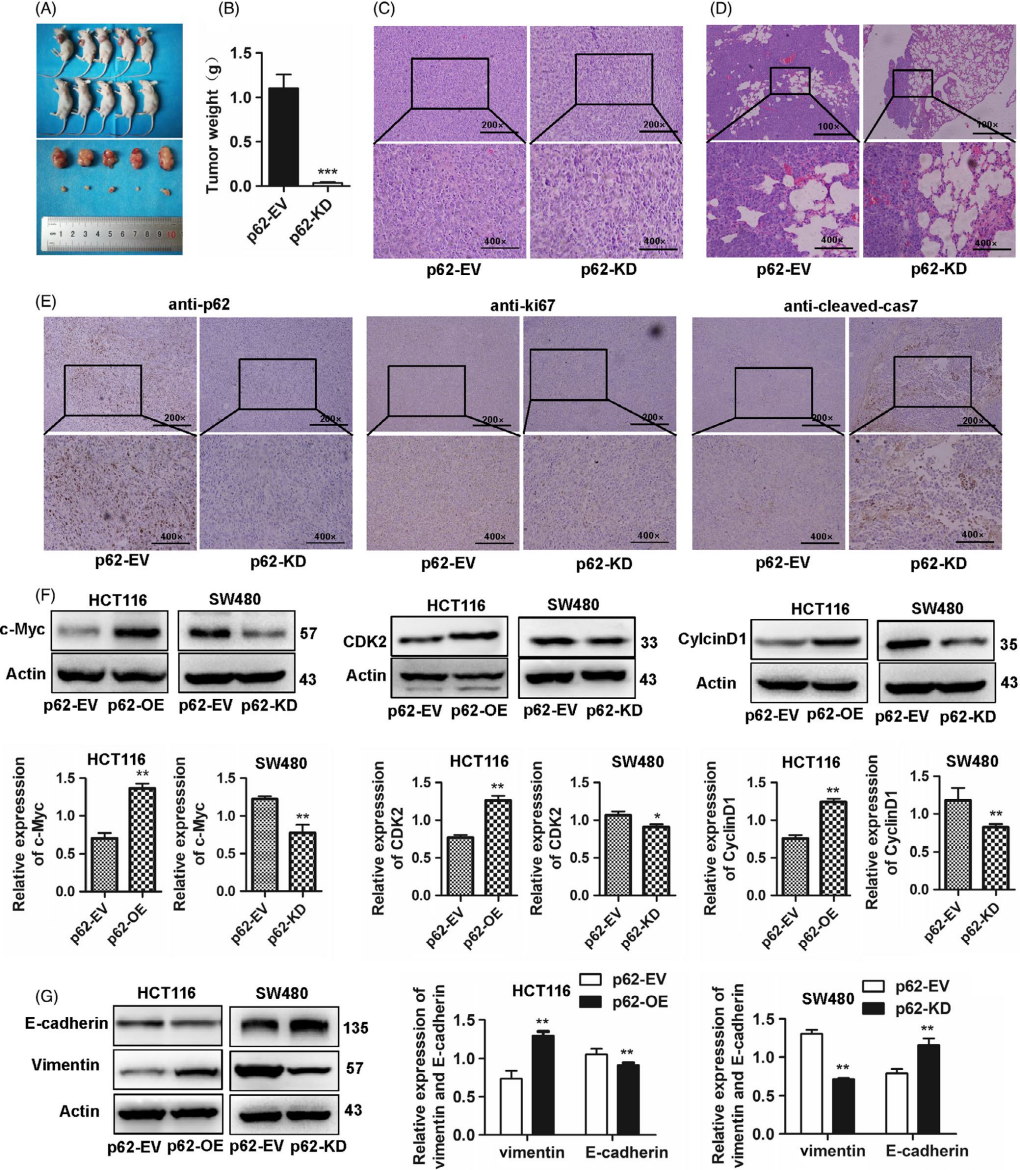
p62 aggravates tumour growth and metastasis in vivo. A, Images of SW480 xenograft tumours show that the knockdown of p62 decelerated growth of SW480 xenografts in nude mice (n = 5) (bottom) compared to that of the controls (top). B, The histogram of tumour weight shows that the knockdown of p62 in SW480 cells formed smaller xenografts compared with those of the control groups (Student's *t* test, ***P < 0.01). C, Representative HE staining of xenograft tumours is shown. D, SW480 (empty vector and p62‐knockdown group) cells were injected into nude mice via the tail vein. Representative HE staining of lung tissue samples is shown. E, Representative images of IHC staining of p62, Ki67 and cleaved‐cas7 in the mouse tumours injected with p62‐knowdown SW480 cells and SW480‐EV cells. F and G, Expression of c‐Myc, CDK2 and cyclin D1 was detected by Western blotting. Expression levels of E‐cadherin and vimentin were detected by Western blotting. Bars represent the standard error of the mean ± SD from three independent experiments. *Represents Student's *t* test *P < 0.05 and **P < 0.01

To explore the in vivo tumorigenic ability of p62, we established a subcutaneous xenograft tumour model. The tumour volumes of nude mice were significantly decreased in the mice injected with p62‐knockdown SW480 cells compared with those injected with SW480‐EV cells (Figure 4A), concomitant with significantly lower tumour weights at the end of the experiment (Figure 4B, *P* < 0.001). We then examined the role of p62 in lung metastasis in vivo. Histological analysis of the tumour metastasis in the lungs revealed that only 20% (1/5) of the mice bearing p62‐knockdown SW480 cells showed lung metastasis after 2 months of induction, while 100% (5/5) of the mice bearing SW480‐EV cells showed lung metastasis (Figure 4C,D). p62 and Ki67 expression levels were lower in mouse tumours injected with p62‐knockdown SW480 cells than those injected with sw480‐EV cells. Cleaved‐cas7 expression levels were higher in mouse tumours injected with p62‐knockdown SW480 cells than those injected with sw480‐EV cells (Figure 4E). The lung volume of nude mice was decreased in the mice injected with p62‐knockdown SW480 cells compared with those injected with SW480‐EV cells (Figure S2). These data indicated that p62 promotes tumour metastasis in CRC.

### 3.5 p62 promotes CRC proliferation and invasion in CRC cells

To further explore the function of p62 on the proliferation of CRC cells, we examined the protein expression levels of c‐Myc, CDK2 and cyclin D1 in human CRC cell lines. The expression of c‐Myc, CDK2 and cyclin D1 protein was increased in p62‐overexpression HCT116 cells, while the expression was decreased in p62‐knockdown SW480 cells (Figure 4F, *P* < 0.05). These data suggest that p62 can promote the proliferation of CRC cells.

Then, we examined the protein expression of the key EMT regulators E‐cadherin and vimentin, which are the key genes in tumour invasion and metastasis, in p62‐overexpression and p62‐knowdown cells. p62 reduced the protein levels of E‐cadherin while enhancing vimentin in p62‐overexpression HCT116 cells. However, knockdown of p62 increased E‐cadherin and decreased vimentin protein expression in SW480 cells (Figure 4G, *P* < 0.01). These data further confirmed the effect of p62 in promoting migration and the invasive abilities of CRC cells through the regulation of EMT.

### 3.6 p62 interacts with the vitamin D receptor and may target the Nrf2‐NQO1 axis through VDR in human CRC cells

**Figure 5:**
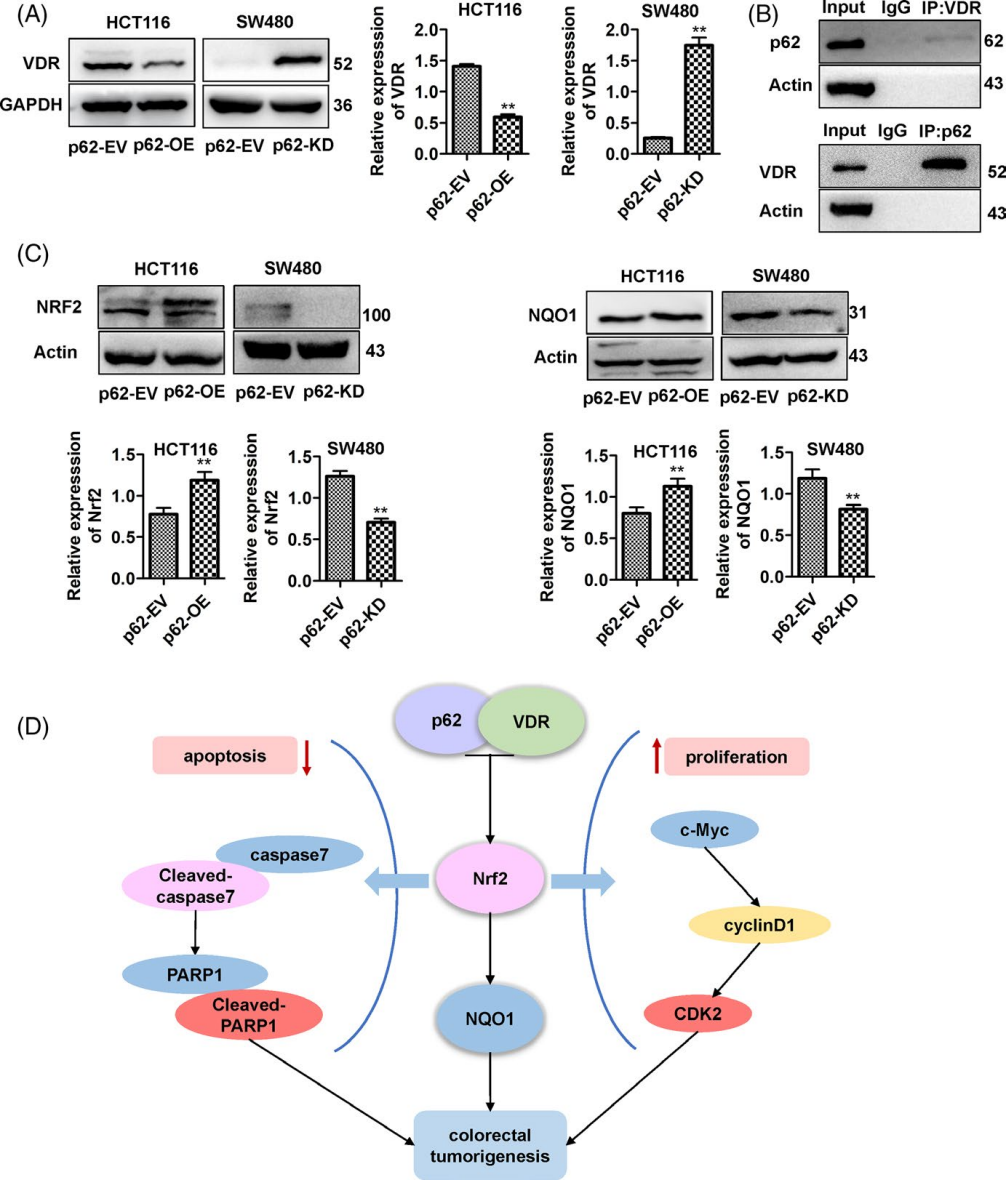
P62 interacts with the vitamin D receptor. A, Expression of the VDR was detected by Western blotting. B, Total lysates from p62‐overexpression HCT116 cells were subjected to IP with VDR Ab or p62 Ab, followed by Western blotting using the indicated antibodies. C, Expression levels of Nrf2 and NQO1 were detected by Western blotting. Bars represent the standard error of the mean ± SD from three independent experiments. *Represents Student's *t* test *P < 0.05 and **P < 0.01. D, Proposed molecular mechanism of p62 in CRC. P62 functions as an oncogene by inhibiting apoptosis and promoting cell proliferation by interacting with the VDR, and p62 may affect the Nrf2‐NQO1 axis through the VDR

To characterize the mechanism of p62 as an oncogene in CRC cells, we measured the protein expression levels of VDR in human CRC cell lines. p62 deficiency enhanced the protein levels of VDR in SW480, and p62 overexpression reduced the protein levels of VDR in HCT116 (Figure 5A, *P* < 0.01). We next examined whether p62 and VDR interact. Co‐IP assays showed that exogenous VDR could coprecipitate with endogenous p62, and exogenous p62 could also coprecipitate with endogenous VDR in p62‐overexpression HCT116 cells (Figure 5B). We also examined the protein levels of Nrf2 and NQO1 and found that the expression of Nrf2 and NQO1 proteins were increased in p62‐overexpression HCT116 cells, while the expression levels were decreased in p62‐knowdown SW480 cells (Figure 5C, *P* < 0.01). Additionally, a piece of the regulatory network of p62 in CRC was elucidated (Figure 5D).

## 4 DISCUSSION

In previous reports, p62 accumulation is frequently observed in a variety of solid tumours. The role of p62 in multiple solid tumours has been demonstrated, highlighting its oncogene effects in ROS generation and cell growth.^6^ Only one study reported that p62 is a prognostic predictor associated with CRC cell proliferation in vitro.^19^ However, studies about the biological effects of p62 in CRC remain unclear. In this study, we first demonstrated that p62 was an oncogene for CRC both in vivo and in vitro and that overexpressed p62 significantly inhibited apoptosis. This may be related to the interaction between p62 and VDR. We first comprehensively analysed the TCGA database and the detection of clinical samples, which proved that p62 is overexpressed in CRC and that the enrichment of p62 predicts poor survival.

Apoptosis is a form of programmed cell death controlled by genes. It is not a phenomenon of self‐injury under pathological conditions but a positive death process to create a suitable living environment for cell growth. Most types of cancer cells show resistance towards apoptosis, and these cells have high proliferative potential.^20^ The effect of p62 on cell apoptosis was further examined by flow cytometry. It was found that the knockdown of p62 in SW480 cells induced noticeable early apoptosis. Caspase‐7 is a member of the caspase family. Activation of caspase‐7 is involved in the execution phase of apoptosis.^21^ Previous studies have demonstrated that caspase‐7 is deficient in CRC.^22^ PARP1 is a poly‐ADP ribose polymerase that participates in DNA repair and apoptosis and can be cleaved by caspase‐7 and caspase‐3. Cleaved PARP is an important marker of apoptosis.^23^ Our study shows that knockdown of p62 in SW480 cells increased the expression of activated forms of cleaved‐caspase‐7 and cleaved‐PARP1. The overexpression of p62 in HCT116 reduced the protein expression of active forms of cleaved‐caspase‐7 and cleaved‐PARP1 (Figure 2E,F). This is the first study to demonstrate that p62 is associated with inhibition of apoptosis in CRC. Then, we used transwell assays and wound healing assays to demonstrate that p62 promotes the invasion and migration abilities in CRC cell lines. p62 also aggravates tumour growth and metastasis in vivo. Therefore, we reasoned that p62 was an oncogene for CRC by inhibiting apoptosis.

We also investigated the effect of p62 on cell proliferation in CRC. C‐Myc is a member of the myc family, which promotes cell proliferation and inhibits differentiation. C‐Myc is often overexpressed in many cancers.^24^ The regulation of the cell cycle mainly depends on cyclins and cell cycle protein kinases (CDKs). Recent studies have shown that cyclins and CDKs are overexpressed in many tumours, closely related to the diagnosis, progression and treatment of tumours.^25^, ^26^ CDK2 plays an important role in the DNA replication of high eukaryotes and the transformation from G1 to s‐phase.^27^ CDK2 overexpression can cross the G1/S limit, shorten the cell cycle and promote cell proliferation. Cyclin D1 overexpression has been shown to be associated with early cancer incidence and tumour progression. Cyclin D1 responds to mitotic growth factors and activates its homologous CDK in the G1 phase.^25^ Our study shows that the expression levels of c‐Myc, CKD2 and cyclinD1 proteins were increased in p62‐overexpression HCT116 cells, while their expression was decreased in p62‐knowdown SW480 cells, demonstrating that p62 also promotes cell proliferation in CRC.

Medical standards have been improving in recent years, but vitamin D deficiency is still a major health problem in the population. The risk factors for developing vitamin D deficiency include low sun exposure, higher geographical latitude,^28^ heavy use of sunscreen^29^ and a diet containing inadequate levels of vitamin D.^30^ In addition to the function of maintaining bone health and metabolism, the liganded VDR functions also involve the regulation of cell proliferation and apoptosis.^31^ In VDR‐null mice, apoptosis of mammary epithelial cells was significantly delayed, supporting the physiological regulation of VDR in apoptosis and glandular development.^32^ In pancreatic islet cells, calcitriol, which is the hormonally active metabolite of vitamin D, preventing TNF/IL‐1‐β/IFN‐ϒ‐induced apoptosis.^33^ In endometrial cancer cells, progesterone and calcitriol combination therapy increased the expression of the vitamin D receptor and inhibited cell proliferation through caspase activation and induction of G0/G1 cell cycle arrest, which is related to the downregulation of cyclins D1.^34^ Vitamin D deficiency and the concomitant loss of vitamin D receptors promotes the growth of breast and prostate cancer cells.^18^ VDR knockout mice showed increased sensitivity to carcinogenic challenge.^30^ Calcitriol can inhibit the growth of breast cancer cell lines, especially in cells expressing ER and VDR. The VDR is a target for breast cancer therapy.^35^ Angeles Duran et al reported that p62 is critical for VDR‐RXR heterodimerization and inhibition of HSCs activation. The VDR signal was damaged after p62 deletion, which enhanced HSCs activation and promoted HCC.^9^ Our study showed that p62 deficiency enhanced the protein levels of VDR in SW480, while p62 overexpression reduced the protein levels of VDR in HCT116. Co‐IP assays showed that p62 interacts with VDR, which we speculated was related to apoptosis and cell proliferation.

Nrf2 is a basic leucine zipper protein that regulates the expression of antioxidant proteins to protect the body from oxidative damage caused by injury and inflammation.^36^ High p62 expression is required to activate Nrf2 and induce c‐Myc to protect HCC cells from oxidative stress‐induced death.^37^ Vitamin D could activate the Nrf2‐Keap1 antioxidant pathway and improve renal disease in diabetic rats.^38^ NQO1 is a conserved target gene of Nrf2 and can be used to monitor the activity of the Nrf2 pathway.^39^ Our research found that the expression levels of Nrf2 and NQO1 proteins were increased in p62‐overexpression HCT116 cells, while the expression levels were decreased in p62‐knowdown SW480 cells.

In conclusion, our findings provide new mechanistic insight into the basic theory of CRC progression and suggest that the blocking of p62 may represent a potential therapeutic strategy for CRC treatment.

## CONFLICT OF INTEREST

The authors have no conflicts to disclose.

## AUTHOR'S CONTRIBUTION

Each author's contribution to the article is as follows: LD, SML and XLL designed the experiments, supervised the study and revised the paper; JZ, SZY, BX, TW and YZ completed the main experiments, wrote the first draft of the paper, prepared the figures and analysed the data; FL and FGR helped with the main experiments; and JJ, HTS and BCZ helped with the preparation of human samples. All authors have reviewed the final version of the manuscript and approve it for publication.
