# Supplementary material for: p62 functions as an oncogene in colorectal cancer through inhibiting apoptosis and promoting cell proliferation by interacting with the vitamin D receptor
Source: Cell Prolif. 2019 Feb 22;52(3):e12585. doi: 10.1111/cpr.12585 (PMC6536406; doi:10.1111/cpr.12585)
Supplement: Supplementary file 1 [file CPR-52-e12585-s001.docx]

**Supplementary information**

**P62 Functions as an Oncogene in Colorectal Cancer through Inhibits Apoptosis and Promotes Cell Proliferation by Interacting with Vitamin D Receptor**

Jing Zhang, Suzhen Yang, Bing Xu, Ting Wang, Ying Zheng, Fei Liu, Fenggang Ren, Jiong Jiang, Haitao Shi, Baicang Zou, Xiaolan Lu, Shemin Lu and Lei Dong

**Supplementary Figures**


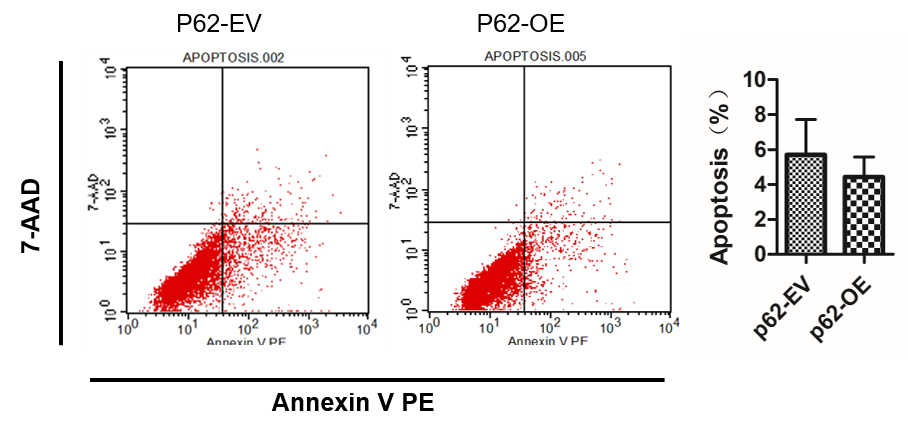


**Supplementary Fig. 1 The apoptosis in p62 overexpressed HCT116 cells.** Overexpression of p62 in HCT116 cells suppressed early apoptosis compared to the control cells, but no statistical difference (p>0.05).


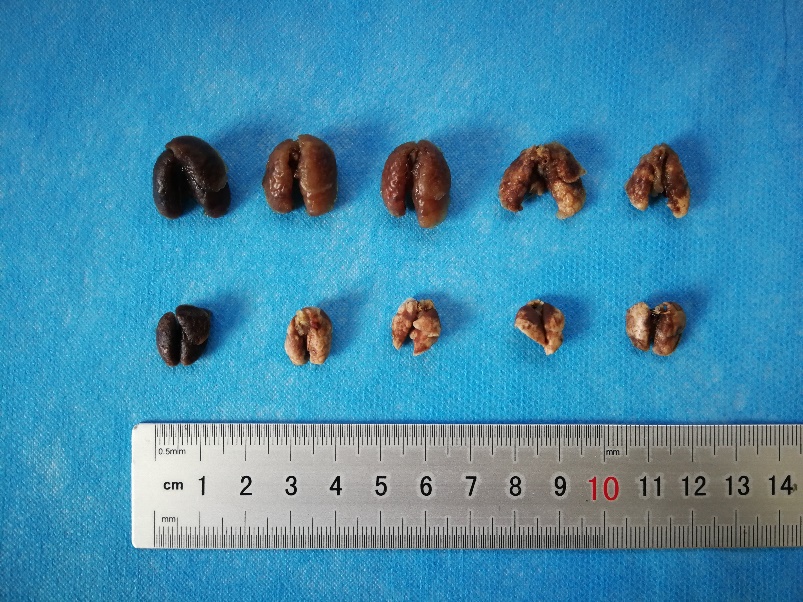


**Supplementary Fig. 2 Images of SW480 lung metastasis.** The lungs volume of nude mice were decreased in the mice injected with SW480-sh p62 cells than in those injected with the SW480-EV cells.
